# Interactions between human orbitofrontal cortex and hippocampus support model-based inference

**DOI:** 10.1371/journal.pbio.3000578

**Published:** 2020-01-21

**Authors:** Fang Wang, Geoffrey Schoenbaum, Thorsten Kahnt

**Affiliations:** 1 Department of Neurology, Feinberg School of Medicine, Northwestern University, Chicago, Illinois, United States of America; 2 National Institutes on Drug Abuse, Intramural Research Program, Baltimore, Maryland, United States of America; 3 Department of Psychiatry and Behavioral Sciences, Feinberg School of Medicine, Northwestern University, Chicago, Illinois, United States of America; 4 Department of Psychology, Weinberg College of Arts and Sciences, Northwestern University, Evanston, Illinois, United States of America; Oxford University, UNITED KINGDOM

## Abstract

Internal representations of relationships between events in the external world can be utilized to infer outcomes when direct experience is lacking. This process is thought to involve the orbitofrontal cortex (OFC) and hippocampus (HPC), but there is little evidence regarding the relative role of these areas and their interactions in inference. Here, we used a sensory preconditioning task and pattern-based neuroimaging to study this question. We found that associations among value-neutral cues were acquired in both regions during preconditioning but that value-related information was only represented in the OFC at the time of the probe test. Importantly, inference was accompanied by representations of associated cues and inferred outcomes in the OFC, as well as by increased HPC–OFC connectivity. These findings suggest that the OFC and HPC represent only partially overlapping information and that interactions between the two regions support model-based inference.

## Abstract 2

Internal representations of relationships between events in the external world can be used to infer outcomes when direct experience is lacking. This study uses fMRI and a sensory preconditioning task to examine how the orbitofrontal cortex and hippocampus contribute to model-based inference.

## Introduction

Decisions can often be made based on direct experience, such as picking among restaurants in which you have dined before. However, in novel situations, direct experience is by definition unavailable, and probable outcomes must be inferred or mentally simulated. For example, how do you decide on whether to try a new restaurant? You may predict the food to be excellent because the new restaurant is affiliated with one of your favorite places (e.g., same chef, owner, hospitality group, neighborhood, etc.). In this case, you are using direct experience with your favorite restaurant and knowledge about the relationship between the two to infer how much you will enjoy the food at the new place. Such inference allows us to predict future outcomes and to make adaptive decisions even when direct experience is insufficient or lacking [1–3].

Inferring future outcomes as in the example above requires the internal representation of relationships between events in the external world [3–10]. This set of relationships is often called a “cognitive map” [11–14] and may consist of a wide range of content relevant to current goals (e.g., spatial, temporal, and associative). In the current study, we focus on the mental representation of associative information that defines the task environment. This can involve not only information about rewards, such as the value of outcomes associated with sensory cues, but also value-neutral information, such as the relationship between different cues. One of the candidate brain regions to implement cognitive maps is the hippocampus (HPC). This proposal was initially inspired by the discovery of place cells [15,16] but was later extended to nonspatial information about objects [17], time [18], and relational knowledge [19]. More recently, rodent and human studies on decision-making have proposed that the orbitofrontal cortex (OFC) also plays a key role in representing and utilizing cognitive maps for organizing behavior directed at obtaining rewards [9,20–24]. The suggestion that both HPC and OFC encode task-relevant information raises questions about how representations in these two areas are related and how OFC and HPC interact to support model-based behavior.

First, do HPC and OFC represent the same information? Although both structures encode many of the same features, neural responses in the OFC appear to be more strongly driven by the biological significance of events [9,25–27]. Thus, it is possible that HPC forms associations between any events, whereas OFC may preferentially encode information about events with biological relevance [28]. Second, if information about the task is represented in both OFC and HPC, where are these representations utilized to infer the likely outcome? The OFC may provide value-related information to the HPC in order to endow relationships between events with motivational significance, or associative information in the HPC might be transferred to the OFC to support inferences about which cues lead to valuable outcomes. Alternatively, input from one area may help to bind individual associations to support inference in the other. Any such integration would require increased coordination between both areas [28].

**Fig 1:**
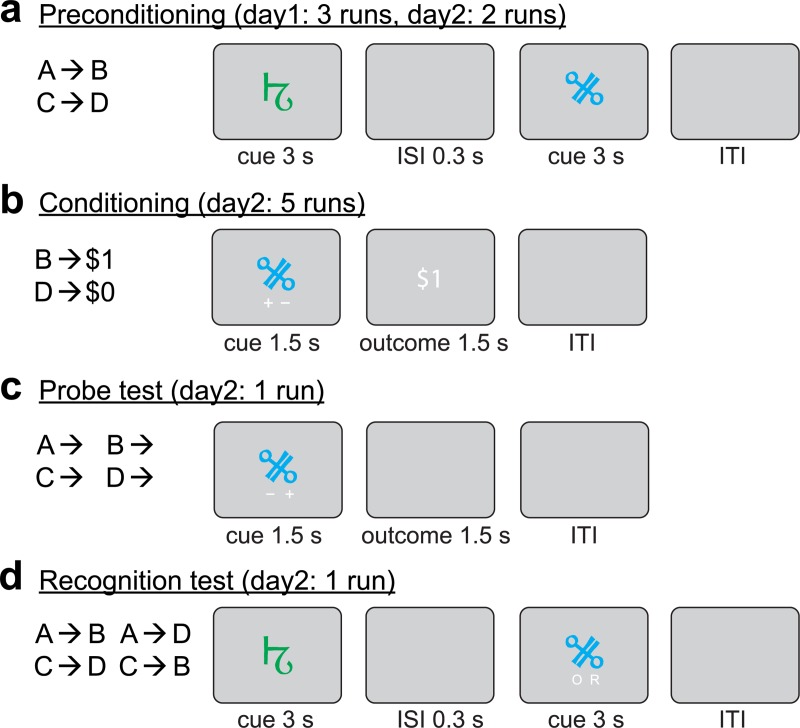
Sensory preconditioning task. (a) Participants learned associations between cue pairs (e.g., A→B) during preconditioning. (b) During conditioning, participants learned associations between the second cue in each pair (B, D) and a monetary outcome ($1, $0). On each trial, one cue was presented, and participants had to predict the outcome associated with the cue by pressing the button corresponding to “+” or “−” presented on the screen. The position of “+” and “−” was randomized across trials. Then, feedback ($1, $0) was presented. (c) During the probe test, participants were asked to make outcome predictions to all cues, but no outcomes were provided. (d) During the recognition test, participants were asked to indicate whether a pair was old (O) or recombined (R). ISI, interstimulus interval; ITI, intertrial interval.

To test these questions, here we used pattern-based functional magnetic resonance imaging (fMRI) in combination with a human version of the sensory preconditioning task. The sensory preconditioning task provides a simple way to experimentally isolate behavior that requires inference from behavior that can be based on direct experience alone [3,29]. It consists of three phases: First, during preconditioning, participants are sequentially presented with pairs of cues (A→B, C→D, **Fig 1A**). Next, during conditioning, participants learn associations between the second cue of each pair and an outcome (B→$1, D→$0, **Fig 1B**). Finally, the outcome prediction based on each cue (A, B, C, and D) is measured in a probe test conducted under extinction conditions (**Fig 1C**). Because cues A and C are never directly paired with an outcome, differential responding to cues A and C in the probe test suggests that participants use associations between A and B (or C and D), and B and $1 (or D and $0), to infer the likely outcomes predicted by cues A and C ($1 and $0, respectively). We refer to B and D as the conditioned cues because they were directly associated with the outcomes, whereas we refer to A and C as the preconditioned cues because they were only associated with the conditioned cues prior to conditioning.

We predicted that if biological relevance were the key factor differentiating information encoded in HPC and OFC, the HPC should acquire associations between the cues during preconditioning, whereas OFC should only become involved when value information is introduced during conditioning and the probe test. To test these predictions, we used a multivoxel pattern analysis (MVPA) to track changes in pattern similarity between paired cues over the course of preconditioning and then to identify where information about the outcomes is represented during the probe test. Moreover, to test whether behavioral and neural responses to the preconditioned cues at the probe test were driven by mediated learning at conditioning [4,30,31] or by model-based inference at the probe test [3,32,33], we examined whether preconditioned cues were reactivated during conditioning and/or whether conditioned cues were reactivated in response to preconditioned cues at the probe test. Finally, if value-related and value-neutral information were distributed across OFC and HPC, responding to preconditioned cues would require binding of this information. We predicted that this would lead to an increase in coordinated activity between both regions. We tested this prediction using a functional connectivity analysis and compared OFC connectivity between trials involving preconditioned and conditioned cues.

## Results

### Behavioral results

#### Preconditioning

**Fig 2:**
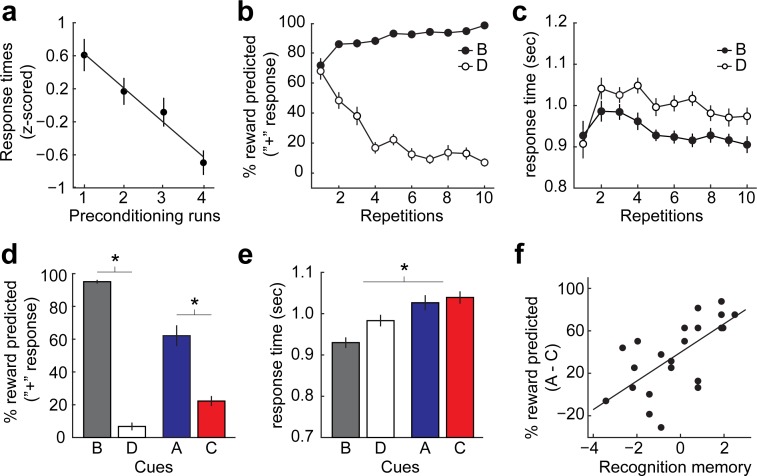
Behavioral responses. (a) Mean response times decreased linearly over preconditioning runs. (b) Mean reward prediction responses (percentage of trials in which participants predicted $1) to cues B and D changed over the course of conditioning. (c) Mean response times of outcome predictions on trials with cues B (minus D) decreased over the course of conditioning. (d) Mean reward prediction responses during the probe test showed stronger responding to cue B than D and to cue A than C. (e) Mean response times during the probe test showed a main effect of cue type, with slower responses to cues A and C compared with cues B and D. (f) Scatter plot depicts significant correlation (*r* = 0.68 *p* = 0.0003) between recognition memory for cue–cue associations (d′) and responding to preconditioned cues (reward prediction responses to cue A minus C) during the probe test. *p < 0.05. Error bars depict ± SEM. Data underlying these plots can be found in **S1 Data**. d′, discrimination sensitivity.

Our experiment included a total of 36 unique visual cues. Thirty-two cues were organized into eight A–B pairs and eight C–D pairs. The four remaining cues were used to form four control cue pairs (E–E). On each trial of the preconditioning phase, participants (*N* = 24) were sequentially presented with pairs of cues (e.g., A→B, C→D, or E→E, **Fig 1A**). Participants were asked to respond with a button press if the second cue was different from the first. The latency of these responses decreased linearly over the course of the preconditioning runs (t[17] = −5.13, *p* = 4.2 × 10^−5^, **Fig 2A**), suggesting that participants acquired information about the predictive relationship between the cues.

#### Conditioning

During the subsequent conditioning phase, participants were presented with conditioned cues B or D, deterministically followed by a reward ($1 after B cues) or no reward ($0 after D cues, **Fig 1B**). To measure learning during conditioning, participants were asked to predict whether the cue presented on each trial would be followed by reward or no reward. Participants were asked to press a left or right button (using the index or middle finger), corresponding to the side of the screen on which “+” or “−” was presented, if they expected the current cue to lead to $1 or $0, respectively. The position of “+” and “−” on the left and right side of the screen was randomized across trials to dissociate outcome expectations from motor-related signals. For each cue type (B and D), we then computed the percentage of trials in which participants predicted a reward and used this percentage as a behavioral measure of reward prediction.

A cue (B versus D) by time (repetitions) ANOVA with repeated measures on prediction responses showed a main effect of cue (F[1,22] = 297.62, *p* = 2.85 × 10^−14^), a main effect of time (F[3.82,84.12] = 8.67, *p* = 9.04 × 10^−6^), and a cue-by-time interaction (F[4.57,100.51] = 42.64, *p* < 0.001, **Fig 2B**). This suggests that reward prediction responses differed significantly between cues B and D and across time, indicating that participants learned the association between the conditioned cues and their outcomes.

In addition, a cue-by-time ANOVA with repeated measures on response times (RTs) showed a main effect of cue (F[1,22] = 31.41, *p* = 1.24 × 10^−5^), a main effect of time (F[3.19,70.08] = 4.95, *p* = 0.003), and a cue-by-time interaction (F[5.8,127.65] = 4.28, *p* = 0.0007, **Fig 2C**), suggesting that compared with the D cues, RTs to the B cues became significantly faster across learning. Taken together, these results show that participants learned the predicted outcomes associated with the conditioned cues over the course of conditioning.

### Probe test

In the probe test, each cue was presented in the same way as in the conditioning phase except that all cues (A, B, C, and D) were presented and no outcomes were delivered (**Fig 1C**). Participants were again asked to indicate whether each cue predicted reward. A cue (conditioned versus preconditioned) by reward (reward versus nonreward) ANOVA with repeated measures on prediction responses showed a significant main effect of reward (F[1,23] = 195.1, *p* = 1.01 × 10^−12^) as well as a cue-by-reward interaction (F[1,23] = 69.14, *p* = 2.2 × 10^−8^, **Fig 2D**), indicating significant reward predictions that were stronger to directly conditioned than preconditioned cues. Importantly, post hoc *t* tests confirmed significant differences in responding to the B cues compared with the D cues (paired *t* test, t[23] = 27.89, *p* = 3.11 × 10^−19^) as well as to the A cues compared with the C cues (paired *t* test, t[23] = 5.69, *p* = 8.55 × 10^−6^). The latter finding demonstrates that participants were able to predict outcomes—reward or nonreward—that were never directly paired with cues A and C.

Similar effects were also evident in RTs (**Fig 2E**), in which a cue (conditioned versus preconditioned) by reward (reward versus nonreward) ANOVA with repeated measures showed significant main effects of cue (F[1,23] = 56.53, *p* = 1.222 × 10^−7^) and reward (F[1,23] = 22.42, *p* = 9.0 × 10^−5^) and a significant cue-by-reward interaction (F[1,23] = 10.16, *p* = 0.0041). The main effect of cue shows that participants took significantly more time to respond to preconditioned cues than conditioned cues, suggesting different cognitive demands for responding to these cues.

To test whether correct responding to the preconditioned cues relied on information about cue–cue associations, we probed participants’ memory of the cue–cue pairs in a post-session recognition test (see **Fig 1D**) and computed the correlation between recognition memory and reward prediction responses to the preconditioned cues. Discrimination sensitivity (d′, reflecting the ability to discriminate between old and recombined cue–cue pairs) was significantly correlated with reward predictions to the A cues compared with the C cues (*r* = 0.68, *p* = 0.0003, **Fig 2F**). This correlation suggests that responding to preconditioned cues is related to cue–cue associations acquired during preconditioning.

### HPC and OFC acquire associations between value-neutral cues during preconditioning

**Fig 3:**
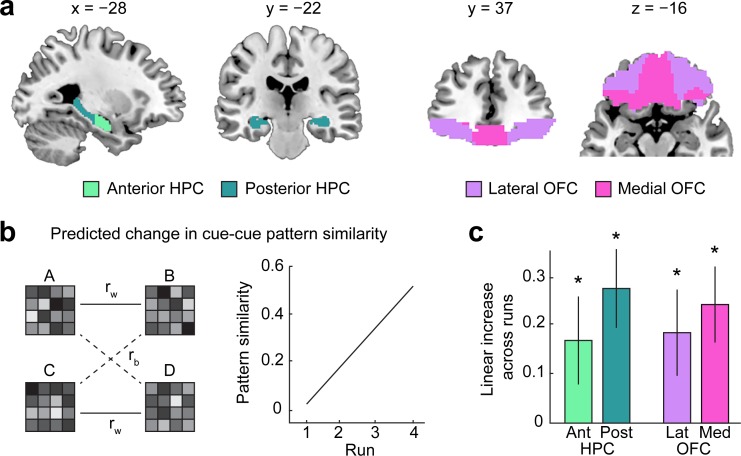
HPC and OFC encode value-neutral cue–cue associations during preconditioning. (a) ROIs in the Ant and Post HPC and Lat and Med OFC. (b) Left: for each ROI, correlations between activity patterns evoked by paired cues (r_w_) and unpaired cues (r_b_) were computed. Right: if a region acquires cue–cue associations during preconditioning, pattern similarity should increase across preconditioning runs. (c) Linear increases in pattern similarity across runs were significant in HPC and OFC. *p < 0.05. Error bars depict ± SEM. Data underlying these plots can be found in **S1 Data.** Ant, anterior; HPC, hippocampus; Lat, lateral; Med, medial; OFC, orbitofrontal cortex; Post, posterior; ROI, region of interest.

Our behavioral results suggest that participants acquired associations between the value-neutral cues during preconditioning. We next focused on the fMRI data to test for neural correlates of this acquisition in the HPC and OFC. Previous work suggests a functional differentiation along the longitudinal axis of the HPC such that anterior HPC is associated with episodic memory encoding, whereas posterior HPC is typically associated with retrieval-related processes [34,35]. In addition, the medial bank of the anterior HPC has been associated with imagining of future episodes [36–38]. Based on this work, we expected a differential involvement of anterior and posterior HPC in the different phases of the sensory preconditioning task and, therefore, analyzed anterior and posterior segments separately (**Fig 3A**). We also analyzed medial and lateral OFC separately because their differential connectivity patterns suggest different functions. Specifically, the lateral OFC receives input from sensory areas, whereas the medial OFC receives input from medial temporal lobe, including HPC [39–42]. In line with these differences in connectivity, previous studies have shown that medial and lateral OFC are primarily involved in the representation of general value and cue- or outcome-specific information, respectively [43–48].

At the neuronal level, the acquisition of cue–cue associations can be evident in increasingly similar neural ensemble responses to the two cues across time [49]. We reasoned that this should be reflected in an increase in the similarity between fMRI activity patterns evoked by the paired cues (e.g., A–B) relative to the unpaired cues (e.g., A–D) across preconditioning (**Fig 3B**). In line with this idea, one-way ANOVAs with repeated measures on the similarity between patterns evoked by paired (minus unpaired) cues did show a significant main effect of time in the anterior and posterior HPC (anterior HPC: F[2.52,55.41] = 4.774, *p* = 0.008; posterior HPC: F[2.52,55.50] = 3.272, *p* = 0.035), as well as the medial and lateral OFC (medial OFC: F[2.58,56.79] = 7.21, *p* = 0.0006; lateral OFC: F[2.74,60.29] = 9.54, *p* = 0.00005). To test whether these main effects were driven by an increase across time, we directly compared pattern similarity between the first and second day of preconditioning. In all areas, pattern similarity for paired (minus unpaired) cues was significantly higher in the second compared with the first day of preconditioning (anterior HPC: t[22] = 3.19, *p* = 0.0021; posterior HPC: t[22] = 2.95, *p* = 0.0037, medial OFC: t[22] = 4.63, *p* = 0.0001; lateral OFC: t[22] = 3.81, *p* = 0.0005). Moreover, mirroring our behavioral measure of cue–cue learning during preconditioning (**Fig 2A**), pattern similarity increased linearly across the four scanning runs (anterior HPC: t[22] = 1.84, *p* = 0.040; posterior HPC: t[22] = 3.34, *p* = 0.0015; medial OFC: t[22] = 3.05, *p* = 0.0029; lateral OFC: t[22] = 2.05, *p* = 0.026, **Fig 3C**). Of note, these increases in pattern similarity could be driven by different types of representational changes. Learning-related changes could occur exclusively for activity evoked by the first or the second cue or for activity evoked by both cues. In any case, these findings suggest that HPC and OFC acquired associations between the value-neutral cues over the course of preconditioning.

In addition, to test for the acquisition of cue–cue associations outside our regions of interest (ROIs), we ran a whole-brain searchlight analysis. This analysis revealed increasing pattern similarity in several areas outside of OFC and HPC, including the visual cortex and insula (**Table 1**).

**Table 1:** Brain regions encoding value-neutral cue–cue associations during preconditioning.

Region	x	y	z	t	*p*
Precentral gyrus	−32	−20	50	6.84	5.05 × 10^−10^
Middle occipital gyrus	34	−94	12	6.43	3.16 × 10^−9^
Insula	−40	0	16	5.33	0.001
Anterior HPC	30	−14	−14	3.8	0.01
Posterior HPC	30	−34	−6	4.11	0.004
	26	−26	−10	3.46	0.028
	22	−38	6	3.4	0.033
Medial OFC	−30	32	−14	3.71	0.047
	−8	60	−10	3.7	0.049
Lateral OFC	30	56	−16	4.59	0.003
	12	68	−10	3.78	0.044
	−28	32	−14	3.75	0.049

Results from whole-brain searchlight (P_FWE_ < 0.05).

Abbreviations: FWE, familywise error; HPC, hippocampus; OFC, orbitofrontal cortex

### OFC represents expected outcomes in response to conditioned cues in the probe test

We next tested how HPC and OFC represented information about the conditioned cues and their associated outcomes during the probe test. For this, we conducted two decoding analyses. The first analysis tested whether there was any information about the conditioned cues and/or their associated outcomes in response to these cues in the probe test. The second analysis aimed to dissociate whether successful decoding in the probe test was driven by representations of the conditioned cues or their associated outcomes ($1 versus $0) independent of cue-specific information.

**Fig 4:**
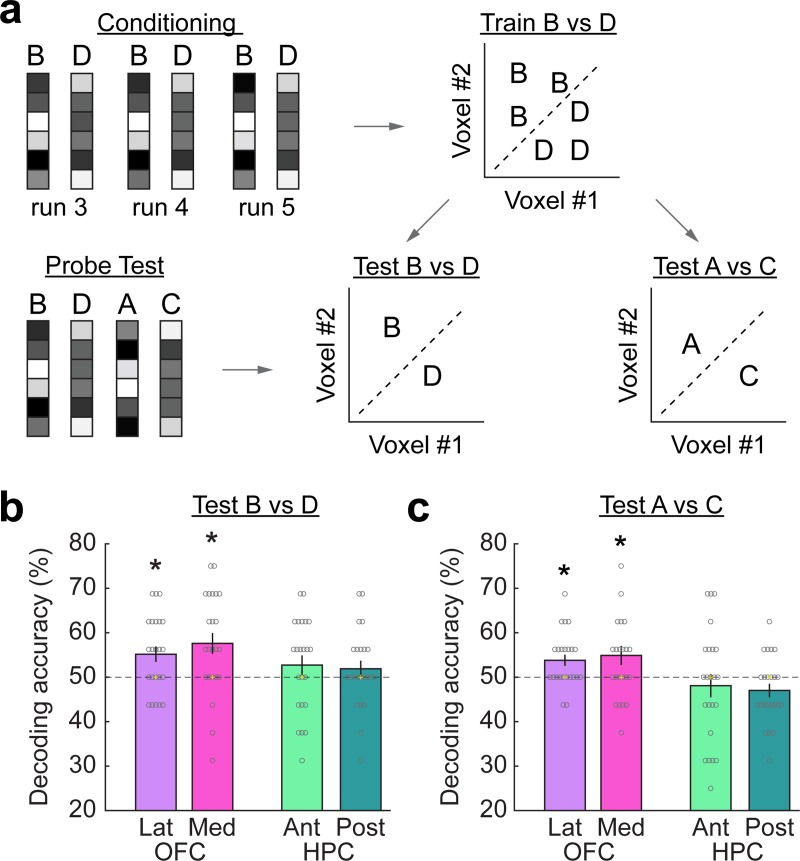
OFC represents expected outcomes in response to conditioned and preconditioned cues. (a) An SVM classifier was trained on ROI activity patterns evoked by cues B versus D during conditioning. The SVM was tested to differentiate between response patterns evoked by cues B versus D as well as cues A versus C in the probe test. (b) Decoding accuracy for cues B versus D was significantly above chance in Lat and Med OFC but not Ant and Post HPC. (c) Mean decoding accuracy for cues A versus C was significantly above chance in Med and Lat OFC but not in Ant and Post HPC. Yellow “+” depicts empirical chance level, and dashed lines depict theoretical chance (50%). Gray circles depict individual data points. *p < 0.05. Error bars depict ± SEM. Data underlying these plots can be found in **S1 Data.** Ant, anterior; HPC, hippocampus; Lat, lateral; Med, medial; OFC, orbitofrontal cortex; Post, posterior; ROI, region of interest; SVM, support vector machine.

For the first analysis we trained a support vector machine (SVM) classifier on fMRI responses evoked by the B versus D cues during the conditioning phase and tested its ability to differentiate fMRI responses to these same cues in the probe test (**Fig 4A**). One-sample *t* tests showed that decoding accuracy in the probe test was significantly above the empirically determined chance level in both the medial and lateral OFC (medial OFC: t[22] = 3.2, *p* = 0.002; lateral OFC: t[22] = 2.91, *p* = 0.004, **Fig 4B**). Decoding accuracy was not significantly above chance in the anterior and posterior HPC (anterior HPC: t[22] = 1.22, *p* = 0.119; posterior HPC: t[22] = 1.02, *p* = 0.159). However, decoding accuracy in the OFC and HPC was not significantly different (t[22] = 1.65, *p* = 0.11).

The second analysis took advantage of the fact that our design included eight sets of cues (i.e., B1, D1, B2, D2, B3, D3, etc.). We trained an SVM classifier to discriminate between activity patterns evoked by all but one set of cues B versus D during conditioning (e.g., cues B8 and D8 were left out) and tested it only on activity patterns evoked by cues B versus D from the left-out cue set (e.g., B8 versus D8). Because this analysis uses different cues for training and testing the classifier, it reveals representations of associated outcome value that are independent of cue-specific information. One-sample *t* tests showed that decoding accuracy was not significantly above chance in medial and lateral OFC (medial OFC: t[22] = 0.72, *p* = 0.239; lateral OFC: t[22] = 0.33, *p* = 0.372). However, a corresponding whole-brain searchlight analysis revealed a cluster in the medial OFC (x = −6, y = 22, z = −16, t[22] = 5.99, p_FWE_ = 0.003, [FWE, familywise error]), indicating a significant contribution from cue-independent representations of associated outcome value in this area. In addition, decoding accuracy was also significantly above chance in left middle temporal gyrus (x = −54, y = −64, z = 22, t[22] = 5.03, p_FWE_ = 0.046; x = −18, y = −76, z = 40, t[22] = 4.69, p_FWE_ = 0.012) and left inferior parietal lobe (x = −54, y = −38, z = 34, t[22] = 4.81, p_FWE_ = 0.036). Taken together, these results suggest that outcomes associated with conditioned cues were represented in the OFC during the probe test.

### OFC represents expected outcomes in response to preconditioned cues in the probe test

We next examined whether OFC also represented expected outcomes in response to the preconditioned cues. For this, we again used two decoding analyses. The first analysis tested whether the preconditioned cues evoked any representation of their paired conditioned cues and/or their associated outcomes in the probe test. The second analysis aimed to dissociate whether successful decoding was driven by representations of the paired conditioned cues (B, D) or their predicted outcomes ($1 versus $0) independent of cue-specific information.

**Fig 5:**
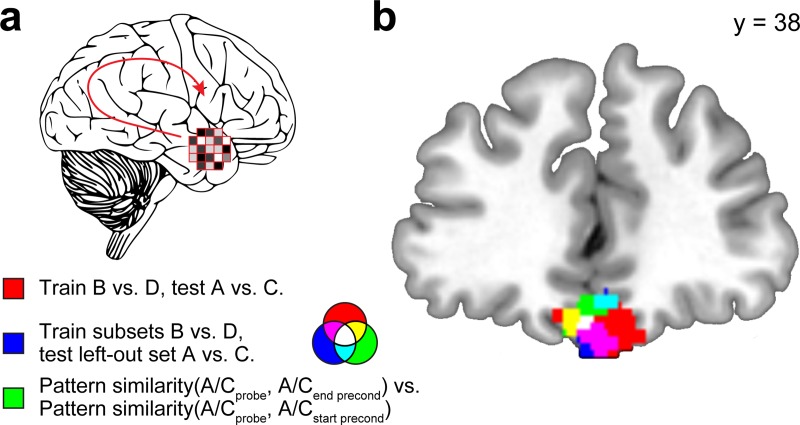
Medial OFC represents expected outcomes and associated conditioned cues in response to preconditioned cues. (a) For each searchlight sphere, we performed three different MVPA analyses. First, an SVM classifier was trained on activity patterns evoked by all sets of cues B versus D during conditioning and was tested to differentiate between response patterns evoked by all sets of cues A versus C during probe test (red voxels in [b]). Second, an SVM classifier was trained on activity patterns evoked by seven sets of cues B versus D during conditioning and was tested to differentiate between response patterns evoked by the left-out set of cues A versus C during probe test (blue voxels in [b]). Third, pattern similarity between cues A/C in the probe test and cues A/C at the end of preconditioning was compared with pattern similarity between cues A/C in the probe test and cues A/C at the beginning of preconditioning (green voxels in [b]). (b) Coronal slice shows overlapping clusters in the medial OFC with significant effects in the three analyses. For illustration, individual maps are thresholded at *p* < 0.005, uncorrected. Whole-brain statistical maps can be viewed at neurovault.org/collections/BMDVXTCY. OFC, orbitofrontal cortex; MVPA, multivoxel pattern analysis; SVM, support vector machine.

For the first analysis we again trained an SVM classifier on cues B versus D during conditioning and then tested whether it could identify the corresponding preconditioned cues A versus C in the probe test. This analysis tests for brain regions in which the preconditioned cues (A, C) activate a representation of their paired conditioned cues (B, D) and/or the outcomes predicted by these cues ($1, $0). One-sample *t* tests showed that decoding accuracy was significantly above chance in both medial and lateral OFC (medial OFC: t[22] = 2.25, *p* = 0.018; lateral OFC: t[22] = 2.96, *p* = 0.004, **Fig 4C**). Decoding was also significantly better in OFC than in the HPC (t[22] = 3.27, *p* = 0.0035), in which decoding accuracy was not above chance (anterior HPC: t[22] = −0.7, *p* = 0.755; posterior HPC: t[22] = −1.91, *p* = 0.965). To test whether additional brain regions represented the paired conditioned cues and/or outcomes in response to the preconditioned cues during the probe test, we performed a whole-brain searchlight analysis. This analysis revealed only one significant cluster in the medial OFC (x = 10, y = 36, z = −24, t[22] = 4.36, p_FWE_ = 0.027, **Fig 5B**), and there was no significant decoding in other brain regions.

The second analysis took advantage of the fact that our design included eight sets of cue–cue pairs (i.e., A1→B1, C1→D1, A2→B2, C2→D2, etc.). We trained an SVM classifier to discriminate between activity patterns evoked by all but one set of cues B versus D during conditioning (e.g., cues B8 and D8 were left out) and tested it only on activity patterns evoked by cues A versus C from the left-out cue set (e.g., A8 versus C8). Because A8 was not directly associated with B1 or B2, etc., this analysis tests for representations of outcomes in response to preconditioned cues independent of specific cue–cue associations. One-sample *t* tests showed that decoding accuracy was significantly above chance in medial OFC (t[22] = 3.22 *p* = 0.002). In contrast, however, decoding accuracy for the left-out cue set was not significant in lateral OFC (t[22] = −1.15, *p* = 0.868), and the difference between medial and lateral OFC was significant (t[22] = 3.91, *p* = 0.0008). These findings were further corroborated by a whole-brain searchlight analysis, which revealed only one significant cluster in the medial OFC, which overlapped with the OFC cluster identified in the first analysis (x = 4, y = 40, z = −26, t[22] = 3.36, p_FWE_ = 0.01, **Fig 5B**). There was no significant decoding in other brain regions. Overall, these results suggest that activity patterns in the medial OFC contained information about the expected outcome ($1, $0) in response to preconditioned cues.

### Preconditioned cues are not reactivated during conditioning

There are two mechanisms generally used to explain the behavioral and neural responses to the preconditioned cues observed here. One is that they are driven by inference (or chaining) at the time of the probe test, with participants recalling the associations between preconditioned and conditioned cues and between conditioned cues and outcomes to infer the outcomes that will likely follow the preconditioned cues. The other is that the preconditioned cues directly acquire value during conditioning by mediated learning or similar mechanisms [4,31]; that is, the preconditioned cues are reactivated when the paired conditioned cues are presented in conditioning, and this reactivation causes them to also acquire value [31].

To distinguish between these possibilities, we examined the neural data for whether the preconditioned cues were reactivated when conditioned cues were presented in conditioning, as required for mediated learning. For this, we compared activity patterns evoked by cues B and D during conditioning with activity patterns evoked by these cues during preconditioning. We reasoned that if the conditioned cues, B and D, were also evoking representations of the paired preconditioned cues, A and C, during conditioning, then the neural pattern in response to them should be more similar to the pattern they evoked at the end (last run) of preconditioning (after the pairings have been acquired) than at the beginning (first run). Contrary to this prediction, the similarity between the activity patterns evoked by cues B and D during conditioning and those evoked by these cues at the beginning and the end of preconditioning was not differentiable (anterior HPC: t[22] = 1.22, *p* = 0.235; posterior HPC: t[22] = −0.22, *p* = 0.826; medial OFC: t[22] = 0.40, *p* = 0.690; lateral OFC: t[22] = 0.22, *p* = 0.825). This suggests that the preconditioned cues were not reactivated during conditioning, making it unlikely that they acquired any value through mediated learning during conditioning in our task [32].

### Preconditioned cues in the probe test evoke representations of conditioned cues in OFC

The above results suggest that preconditioned cues were not reactivated during conditioning. However, they do not provide positive evidence for the alternative hypothesis—namely, that responding to preconditioned cues is based on inference at the time of the probe test. This hypothesis predicts that neural representations of paired conditioned cues are evoked in response to the preconditioned cues at the time of probe test such that their likely outcomes can be inferred. To directly test whether cues A and C evoked representations of paired cues B and D, respectively, we conducted an additional analysis. Similar to the analysis presented above, we reasoned that if representations of conditioned cues were evoked in response to the preconditioned cues in the probe test, then activity patterns to cues A and C during the probe test should be more similar to those at the end of preconditioning (last run), after learning of the associations with B and D, than at the beginning of preconditioning (first run), prior to learning. Importantly, because cues had not acquired any value at the time of preconditioning, this analysis is independent of any value confounds.

In line with this prediction, we found that the activity patterns evoked in OFC by the preconditioned cues in the probe test were more similar to representations of these cues at the end compared with the beginning of preconditioning (medial OFC: t[23] = 3.73, *p* = 0.001; lateral OFC: t[23] = 2.32, *p* = 0.029). No significant differences were found in the HPC (anterior HPC: t[23] = 1.04, *p* = 0.312; posterior HPC: t[23] = 0.71, *p* = 0.483). A corresponding searchlight analysis revealed a cluster in the medial OFC, which overlapped with the clusters that represented expected outcomes in response to the preconditioned cues (x = −4, y = 38, z = −20, t[23] = 3.70, **Fig 5B**). These findings suggest that preconditioned cues evoked value-neutral representations of the associated conditioned cues in the OFC at the probe test.

Because the last run of preconditioning occurred on the same day as the probe test, the higher correlation between activity patterns evoked by cues in this run could be driven by scan day–related effects. To rule out this possibility, we performed a control analysis in which scan day–related correlations (e.g., scan day–related differences in correlations between unrelated cues A and C) were subtracted from the correlations of interest, effectively removing any scan day–related confounds. This control analysis revealed significant effects in the medial OFC (t[23] = 2.05, *p* = 0.026) but not lateral OFC (t[23] = 1.29, *p* = 0.105). Again, no differences were found in the HPC (anterior HPC: t[23] = 0.71, *p* = 0.243; posterior HPC: t[23] = 0.75, *p* = 0.232).

### OFC–HPC connectivity facilitates model-based inference

**Fig 6:**
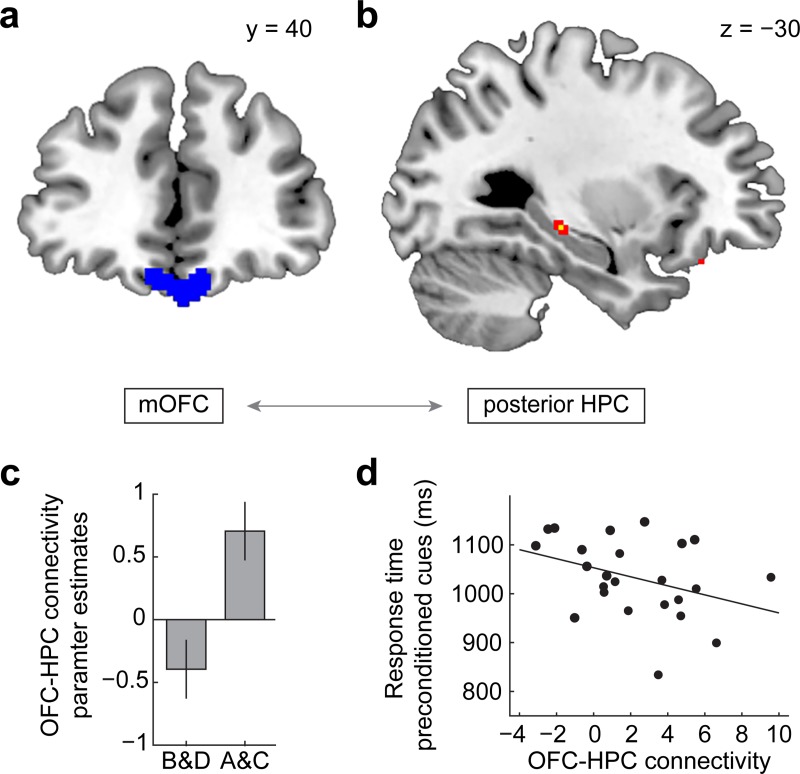
OFC–HPC connectivity facilitates model-based inference at the time of decision-making. (a) Illustration of the mOFC seed region used in the PPI analysis, which was defined as the mOFC cluster identified in the searchlight-based decoding analysis (*p* < 0.005, uncorrected). (b) Cluster in the posterior HPC with significantly greater OFC connectivity during probe test trials with preconditioned cues compared with conditioned cues. For illustration, map is thresholded at *p* < 0.005 (red) and *p* < 0.001 (yellow), uncorrected. (c) Mean functional connectivity extracted for illustration from the posterior HPC cluster for preconditioned cues (A and C) and conditioned cues (B and D). (d) HPC–OFC functional connectivity was negatively correlated with response time during inference (r = −0.36, *p* = 0.040). Error bars depict ± SEM. Data underlying these plots can be found in **S1 Data.** The whole-brain statistical map can be viewed at neurovault.org/collections/BMDVXTCY/images/306228/. HPC, hippocampus; mOFC, medial OFC; OFC, orbitofrontal cortex; PPI, psychophysiological interaction.

The results described above suggest that preconditioned cues evoked representations of conditioned cues as well as their associated outcomes in overlapping areas of the OFC at the time of the probe test. However, cue–cue associations were also represented in the HPC during preconditioning, and previous work indicates that HPC projections to the OFC are critical for establishing integrated representations of task state in the OFC [50]. We therefore hypothesized that HPC–OFC connectivity might support model-based inference. To test this prediction, we implemented a psychophysiological interaction (PPI) analysis [51] with medial OFC as the seed region (**Fig 6A**) and compared connectivity between trials involving preconditioned and conditioned cues. We found that functional connectivity between medial OFC and posterior HPC was significantly higher on trials with preconditioned cues compared with trials involving conditioned cues (x = −30, y = −26, z = −8, t[23] = 3.68, p_FWE_ = 0.042, **Fig 6B** and **6C**). No other brain regions showed this connectivity effect. Moreover, the connectivity modulation between OFC and HPC was significantly correlated with RT for preconditioned cues (r = −0.36, *p* = 0.040, **Fig 6D**) but not conditioned cues (r = −0.25, *p* = 0.123), suggesting that HPC–OFC connectivity may selectively facilitate model-based inference.

## Discussion

In this study, we used a sensory preconditioning task and fMRI to examine the role of OFC and HPC in supporting outcome predictions based on cues that were never directly paired with reward. Pattern-based fMRI analyses showed that although both OFC and HPC acquired cue–cue associations during preconditioning, only OFC contained information about expected outcomes in response to conditioned cues. In the probe test, participants made reward prediction responses to preconditioned cues, which were accompanied by representations of conditioned cues as well as their associated reward in OFC. In addition, connectivity between HPC and OFC was selectively increased on trials involving preconditioned cues.

During preconditioning, both HPC and OFC acquired associations between the value-neutral cues. Whereas HPC has long been known to represent associative information [16,17,19,52], OFC has historically been considered to primarily process information with biological significance [9,53–58]. However, our current findings suggest that OFC acquired associations between cues even before they were endowed with value. This mirrors recent findings in rats, showing that OFC ensemble patterns form associations between value-neutral cues during preconditioning [49], and extends earlier observations that OFC encodes value-neutral features of expected outcomes [24,43,50,59]. It should be emphasized that participants were not aware of monetary outcomes in the preconditioning phase, and thus, these findings are not contaminated by encoding of reward information. Although a role for social value originating from experimenter demands cannot be ruled out, these findings suggest that both OFC and HPC can acquire associative information even when that information is of limited or at least uncertain biological significance.

OFC and HPC differed in their representation of reward outcomes associated with the conditioned cues during the probe test. Such information was present in the OFC but not in HPC, at least at the level of our measure. This suggests that information in OFC may differ from that in HPC by information content [60,61] such that OFC represents information with and without biological significance [22], whereas HPC is less driven by value-related information in this setting. Alternatively, this may suggest that HPC is primarily involved in the acquisition of information, which can then be used by different brain areas, such as OFC, for establishing task representations for guiding behavior [62,63], and as a result, it is less engaged by the subsequent use of this information.

Regarding the question of when and how preconditioned cues acquired reward information, our findings are more compatible with the idea that participants inferred the outcome at the time of the probe test in response to preconditioned cues. Although some studies indicate that cached values can spread to preconditioned cues during conditioning via mediated learning or similar mechanisms [4,30,31,64], we saw no evidence that the preconditioned cues were reactivated during conditioning. As noted earlier, mediated learning requires such reactivation, whereas inference, though not incompatible with reactivation, does not require it. The lack of reactivation here is consistent with proposals that such reactivation is primarily favored when the conditioned cue is presented together or even before the preconditioned cue (i.e., B→A). Our use of the opposite procedure (i.e., A→B) is thought to favor inference. Accordingly, work using a similar A→B training procedure in rats has shown that preconditioned cues do not support conditioned reinforcement, the gold standard for assessing cached value [32]. Furthermore, inactivation of the OFC in the probe test in this setting abolishes responding to the preconditioned cues in rats without affecting responding to the conditioned cues [3]. If preconditioned cues were to acquire cached value in the conditioning phase, as if they were present and directly paired with reward, then OFC inactivation would not abolish adaptive responding in the probe test, given that OFC is not necessary for behavior that is based on directly learned cue–outcome associations [65–67]. Finally, we found evidence that preconditioned cues evoked representations of conditioned cues in the probe test. Such representations are necessary for model-based inference, but they are not predicted by or necessary for mediated learning [3]. Based on these reasons, we conclude that in our study, participants’ responses to preconditioned cues were based on model-based inference at the time of decision-making. Given that overlapping areas of OFC represented the conditioned cues and the inferred outcomes in response to the preconditioned cues at the time of the probe test, it is tempting to speculate that inference occurred within the OFC. Although these individual pieces of information are likely represented by different neuronal populations within the OFC, their presence is reminiscent of a set of associative representations that could be used to carry out the inference [20,62].

Whereas the HPC did not exhibit representations in the probe test similar to those in OFC, we found that inference was associated with increased HPC–OFC connectivity. In addition, connectivity was linked with faster responses to preconditioned cues, further suggesting that interactions between HPC and OFC may facilitate model-based inference. This is consistent with findings from human and animal studies on transitive inference. In transitive inference tasks, subjects are trained to learn a set of overlapping stimulus pairs (e.g. A > B, B > C, C > D, D > E) and are tested on probe trials that require transitive inference (B > D?) [68]. The sensory preconditioning task is structurally similar but requires inference about reward outcomes to guide decisions. Studies on transitive inference have shown that activity in HPC and ventromedial prefrontal cortex (vmPFC) is correlated with transitive inference [68–70] and that lesions to these regions selectively impair animals’ performance on trials requiring transitive inference [68,71]. Our functional connectivity findings are compatible with the idea that HPC projections to the OFC bind isolated pieces of associative information in the OFC to form an integrated representation of the task that can be used for inference. This is in line with recent rodent work showing that HPC input to the OFC is necessary for establishing task state representations in the OFC [50]. Moreover, it is consistent with reports that amnesic patients with bilateral HPC damage fail to form holistic representations of future events and instead represent fragmented elements of the experience [72,73]. The HPC–OFC connectivity result is also consistent with the idea that HPC is important for reinstating representations of cortical activity that occurred during learning [74,75].

Although it is difficult to interpret negative results, a role of HPC in binding and reinstating memory representations in cortical areas may explain the lack of evidence for cue–cue associations in the HPC during the probe test. Specifically, HPC may only encode cue–cue associations during the initial learning stage, whereas this information is stored and consolidated in the OFC. When cue–cue associations are required for outcome predictions in response to preconditioned cues in the probe test, the HPC may then reinstate or bind representations in the OFC without itself representing these associations. This is consistent with recent evidence that HPC is primarily involved in the acquisition of information, which can then be used by different brain areas to guide behavior [76,77].

In summary, our findings bridge and extend previous work on the role of OFC and HPC in model-based behavior. We found that the OFC and HPC represented partially overlapping information, such that HPC acquires value-neutral information during initial learning, whereas the OFC contains representations including but not limited to information with biological significance or value. Most importantly, our results suggest that model-based inference in the OFC is supported by its interactions with the HPC.

## Materials and methods

### Ethics statement

The study protocol was approved by the Northwestern University Institutional Review Board (STU00202875), and the experiments were conducted according to the principles expressed in the Declaration of Helsinki. All subjects gave written informed consent to participate.

### Subjects

Twenty-nine healthy human participants with no history of psychiatric illness (12 male; age 19–32 years; mean ± SEM, 24.42 ± 0.67 years) participated in this study. Data from five participants were excluded from all analyses because their behavioral performance in the last conditioning run was not significantly above chance. One participant was excluded from the analyses of the preconditioning data because of technical problems during the preconditioning scan. One participant was excluded from all analyses related to the conditioning phase because of technical problems during the conditioning scan.

### Stimuli and experimental procedures

Cues consisted of 36 abstract visual symbols. Thirty-two symbols were randomly selected and grouped into 16 pairs for each participant, of which half served as A–B pairs and half served as C–D pairs. Thus, there was a total of eight sets of A–B and C–D cue pairs. The four remaining symbols were used to form four control pairs (E–E) in which the same symbols were presented twice in a row (E1–E1, E2–E2, etc.). The two symbols in a pair were presented in different colors (blue = first and green = second, counterbalanced across participants). Each symbol was shown against one of two scene background images: a forest or a field of flowers. Two odors, fir needle oil (Lhasa Karnak Herb Company) and plum blossom (Aroma Workshop) were delivered to participants’ noses through a custom-built, computer-controlled olfactometer [24].

The study was completed on two consecutive days, and the MRI data were acquired during all runs of preconditioning, conditioning, and probe test. On the first day, participants were instructed to learn target and control cue pairs (target: A→B, C→D; control: E→E) in three preconditioning runs. The cues in a pair were presented one after another for 3 s each, separated by a delay of 300 ms. A fixation cross appeared between trials for a jittered duration between 3 and 11 s. To ensure attention to the cue pairs during preconditioning, participants were instructed to memorize the cue pairs, press a button if the second symbol was different from the first, and withhold a response if the two symbols were identical. In the first run of preconditioning, each cue pair was repeated three times in a row. In the remaining preconditioning runs, the order of cue pairs was randomized. Because of these differences in stimulus presentation, fMRI data from the first preconditioning run were excluded from all analyses. On the second day, participants performed two additional runs of preconditioning. Participants were not informed about the monetary associations until the conditioning phase.

Next, participants performed five runs of conditioning, during which the second cue (cues B and D) of each pair was presented individually for 1,500 ms. Participants were instructed to make predictions about the monetary outcome ($0 or $1) they expected following the cue. If they expected the cue to be followed by $1, they were asked to select “+,” and if they expected $0, they were asked to select “−.” Participants made their prediction by pressing a button with the index or middle finger or their right hand corresponding to the position of “+” and “−” on the screen. The position of “+” and “−” on the screen was randomized across trials to dissociate motor responses from reward predictions. A $1 or $0 outcome feedback was presented for 1,500 ms immediately after the cue if participants responded within 1,500 ms. Otherwise, “too slow” was displayed. Each cue–outcome association was repeated twice in each run in pseudorandomized order, resulting in 10 repetitions total.

In the following probe test, all cues were presented individually without any feedback. Each cue was presented four times in pseudorandomized order. Participants were instructed to make reward predictions for each cue as they did during conditioning. They were instructed to use the cue–cue associations to infer the outcomes associated with the preconditioned cues (A, C). The durations of cue and feedback presentation, as well as the interval between trials, were exactly the same as they were during conditioning. For half of the cues, the same background (pictures and odors) as the one used during preconditioning was presented during the probe test, whereas the other background was presented for the rest of the cues. Because this manipulation had no effect on behavioral performance (*p*-values > 0.71) and fMRI-based decoding accuracies (*p*-values > 0.12), we collapsed data across both conditions.

Following the probe test, participants were tested for their memory of cue–cue associations in a recognition task. Participants were presented with the original cue pairs as well as recombined pairs consisting of cues belonging to different pairs. Pairs were presented sequentially as was done during preconditioning, and participants were asked to indicate whether a pair was old or recombined after the second cue was presented.

### fMRI data acquisition

The MRI data were acquired at the Northwestern University Center for Translational Imaging (CTI) using a 3T Siemens PRISMA scanner equipped with a 64-channel head coil. Functional images were acquired with an echoplanar imaging (EPI) sequence with the following parameters: repetition time (TR), 2 s; echo time (TE), 22 ms; flip angle, 90°; slice thickness, 2 mm; no gap; number of slices, 58; interleaved slice acquisition order; matrix size, 104 × 96 voxels; field of view, 220; multiband factor, 2. In order to minimize susceptibility artifacts in the OFC, the acquisition plane was tilted approximately 25° from anterior commissure (AC)–posterior commissure (PC) line [78]. We collected partial brain volumes without coverage of the dorsal portion of the parietal lobes. The number of EPI volumes in each of the preconditioning runs ranged from 343–450. Each conditioning run consisted of 132 EPI volumes, and the probe test included 502 EPI volumes. We also acquired 10 whole-brain EPI volumes for each participant, which had the same parameters as described above except with 95 slices and a TR of 5.25 s. These volumes were used to improve coregistration of the EPI time series data. In addition, anatomical images were collected for spatial normalization using a MPRAGE sequence with 1-mm isotropic voxels.

In order to monitor breathing during scanning, a respiratory effort band (BIOPAC Systems, Goleta, CA) was affixed around the participant’s torso. Breathing traces were recorded using PowerLab equipment (ADInstruments, Dunedin, New Zealand) at a sampling rate of 1 kHz. Breathing traces were smoothed using a moving window of 250 ms, high-pass filtered (cutoff, 50 s), z-scored within each run, down-sampled to 0.5 Hz, and included as nuisance regressors in all fMRI data analyses.

### fMRI data preprocessing

Preprocessing was performed using Statistical Parametric Mapping (SPM12) software (www.fil.ion.ucl.ac.uk/spm/). For each participant, all functional EPI images across all fMRI runs were aligned to the first acquired image to correct for head motion during scanning. The 10 whole-brain EPI images for each participant were also realigned and then averaged. Both the functional EPI images (using the mean EPI) and the mean whole-brain EPI were coregistered to the anatomical image for each participant. Spatial normalization was conducted in two steps. First, we normalized each participant’s anatomical image to the Montreal Neurological Institute (MNI) space using the six-tissue probability map provided by SPM12. The resulting deformation fields were then applied to the EPI images to transform them into MNI space. Finally, the normalized functional EPI images were spatially smoothed with a Gaussian kernel that was 6 × 6 × 6 mm. For all MVPA analyses, the motion-corrected functional images were resliced, smoothed with a Gaussian kernel that was 2 × 2 × 2 mm, and analyzed in native space.

All first-level general linear model (GLM) analyses of the fMRI functional data described below were conducted using SPM12 and included the following nuisance regressors: the smoothed, normalized, and down-sampled sniff trace; the six realignment parameters (three translations, three rotations) calculated for each volume during motion correction; the derivate, square, and the square of the derivative of each of the realignment regressors; the absolute signal difference between even and odd slices and the variance across slices (to account for fMRI signal fluctuation caused by within-scan head motion); the squares, derivatives, and squared derivatives of these two within-volume measures; and additional regressors as needed to model out individual volumes with particularly strong head motion.

### ROI definition

The HPC mask was created by manually tracing on the MNI template brain based on anatomical criteria described in previous studies [79]. In brief, the anterior border of the HPC was defined by a thin line of white matter separating the HPC and the amygdala. The posterior border was defined as where the gray matter disappears near the lateral ventricle. The HPC was further segmented into anterior and posterior portions based on the uncal apex landmark [34]. Masks for the medial and lateral OFC were taken from a previous study, which used unsupervised clustering of resting-state connectivity to parcellate the OFC [80]. For MVPA analysis, ROIs were inverse-normalized into native space.

### MVPA to test for acquisition of cue–cue associations during preconditioning

To investigate whether HPC and OFC acquired information about cue–cue associations during preconditioning, we conducted a pattern-based similarity analysis on minimally smoothed functional images in native space. The second and third run from the first day and both runs from the second day were included in the analysis of the preconditioning data (the first run from the first day was excluded because it had a different trial sequence). We first estimated a first-level GLM with four regressors of interest: cues A, B, C, and D. Regressors were created by convolving the onsets and durations of each cue with the hemodynamic response function, and t-maps were computed based on the resulting parameter estimates. For each preconditioning run, we computed the similarity (Fisher’s Z transformed Pearson’s correlation) between multivoxel response patterns evoked by cues in a given pair (A and B, C and D) and cues not belonging to a pair (A and D, C and B). This was done separately for each ROI. To control for unspecific changes in pattern similarity across runs (e.g., effects of attention to the first cue in each pair), within each run, we subtracted the pattern similarity between unpaired cues (A and D, C and B) from pattern similarity between paired cues (A and B, and C and D). Note that because of short interstimulus intervals between paired cues, the pattern similarity between the first and second cue in a pair is confounded by temporal proximity. However, the correlation driven by temporal proximity should be constant across runs, and thus, pattern similarity increases across runs should not be contaminated by temporal proximity and only capture effects related to the acquisition of cue–cue associations.

### MVPA to test for outcomes in response to conditioned and preconditioned cues

To test for representations of outcomes associated with conditioned cues (i.e., B→$1, D→$0) in the probe test, we used an SVM classifier with feature selection. For each subject, we first estimated separate GLMs for the last three conditioning runs and the probe test. We focused on the last three runs of conditioning because behavioral performance stabilized after the first two runs (**Fig 2B**), suggesting that learning was completed. Specifically, pairwise comparisons in ascending order showed that behavioral performance (percentage reward predicted on B minus D trials) in the fourth repetition was significantly higher than that in the third repetition (t[22] = −4.93, *p* = 6.18 × 10^−5^) but that there were no significant differences between adjacent repetitions starting from the fifth repetition (t[22] = 0.15, *p* = 0.88). The conditioning GLMs included two regressors of interest coding for the onset of cues B and D, along with regressors modeling the onset of the outcome, and left and right button presses. The probe test GLM included 32 regressors of interest coding for the onset of each symbol, along with regressors modeling the onset of left and right button presses. These models generated maps of parameter estimates for each regressor of interest for which t-scores were computed.

The decoding analysis was conducted in HPC and OFC subregions separately. We used the SVM provided by the LIBSVM implementation with a linear kernel and a default c = 1 [81]. For each ROI and subject, we trained an SVM classifier to classify activity patterns evoked by cues B versus D during the conditioning phase and tested it on activity patterns evoked by cues B versus D, as well as cues A versus C, during the probe test phase. Note that we never trained any classifier on data from the probe test.

We used a leave-one-subject-out cross-validated feature selection procedure to determine the optimal number of voxels to be included in the SVM. For all but one left-out subject, we computed the t-value by comparing fMRI responses between cues B and D across the last three runs of conditioning. Then, the voxels were rank ordered based upon the absolute t-value. Starting from the 40 best voxels with the largest absolute t-values for the OFC and the 20 best voxels for the HPC, we trained several SVM models, and these models were then tested on the probe test data of all but the left-out subject. We then repeated this procedure and included an additional 40 and 20 voxels for OFC and HPC, respectively. This resulted in an average classification accuracy for every number of included voxels. The included number of voxels that led to the highest average decoding accuracy in the training sample was defined as the optimal voxel number and used to perform the decoding analysis in the left-out subject. This nested feature selection procedure was repeated for each subject left out, and decoding accuracies from the left-out participants were used to determine the final decoding accuracy. The scripts implementing this analysis can be viewed at github.com/fangw12/Sensory_Preconditioning.

In addition, we performed a cross-pair classification analysis in which we trained an SVM classifier on activity patterns evoked by all but one set of cues B versus D during conditioning (e.g., B1 versus D1, B2 versus D2, etc.) while leaving one set of cues out (e.g., B8 and D8). We then tested this SVM classifier on activity patterns evoked during the probe test by cues B versus D, as well as A versus C from the left-out cue set (e.g., B8 versus D8, and A8 versus C8). This was repeated eight times, each time with one cue set left out, and the results were averaged.

We also conducted whole-brain searchlight-based decoding analyses in order to identify additional brain areas in which conditioned cues and inferred outcomes were represented in the probe test. The procedure of these analyses was the same as the ROI-based approach described above but did not involve feature selection. The radius of the searchlight sphere was 8 mm, resulting in 251 voxels.

### MVPA to test for reactivation of preconditioned cues during conditioning

To test for a reactivation of the preconditioned cues A and C in response to cues B and D during conditioning, we conducted the following pattern similarity analysis. For each subject, we first computed template patterns evoked by cue B during conditioning within the OFC and HPC ROIs. We next computed correlations between these template patterns and activity patterns evoked by cue B in the first and last run of the preconditioning phase. The resulting correlations were compared between the first and last run. The same steps were performed for cue D, and results were averaged across B and D. We reasoned that if activity patterns evoked by cue B in conditioning contained information about cue A, these must have been acquired during preconditioning. Accordingly, the activity patterns to cue B in conditioning should be more like those to cue B in the last compared with the first run of preconditioning—that is, after versus before B could have acquired any association with cue A.

### MVPA to test for reactivation of conditioned cues in response to preconditioned cues at the probe test

To test for a representation of the conditioned cues B and D in response to the preconditioned cues A and C in the probe test, we conducted a comparable pattern similarity analysis. For each subject, we first computed template patterns evoked by cue A during the probe test within the OFC and HPC ROIs. We next computed correlations between these template patterns and activity patterns to cue A in the first and last run of preconditioning. The resulting correlations were compared between the first and last run. The same steps were performed for cue C, and results were averaged across A and C. We reasoned that if activity patterns evoked by cues A and C in the probe test contained information about cues B and D, these must have been acquired during preconditioning. Accordingly, the activity patterns to A and C in the probe test should be more like those to cues A and C during the last run compared with the first run of preconditioning—that is, after versus before they could have acquired any association with cues B and D. To search for reactivation outside our ROIs, we performed a corresponding searchlight analysis.

### PPI analysis

To examine changes in OFC connectivity during inference trials, we conducted a PPI analysis using the gPPI toolbox [51]. Specifically, for each participant, we estimated a PPI model with preconditioned versus conditioned cues as the psychological factor and fMRI activity in the medial OFC as the physiological variable. The seed region was defined as the medial OFC cluster identified in the searchlight-based decoding analysis (at *p* < 0.005, uncorrected). The model also included regressors coding for the onsets of left and right button presses as well as the nuisance regressors described above.

### Group-level statistical analysis

To examine changes in pattern similarity across runs, for each ROI we computed a one-way ANOVA with repeated measures on the z-scored correlation difference to test for differences between runs. For significant main effects, we then directly compared pattern similarity between the first and second half of the preconditioning data using paired *t* tests. Finally, to test whether pattern similarity increased linearly across runs, we tested whether the linear increase across runs was significantly different from zero using one-sample *t* tests.

To test for representations of outcomes in the probe test in response to cues B versus D or A versus C, we performed one-sample *t* tests on decoding accuracies (minus empirical chance level, see below) in each ROI. In addition, to identify brain regions outside our ROIs that represented cue–outcome associations in response to cues A versus C in the probe test, we conducted voxelwise one-sample *t* tests on normalized (MNI space) and smoothed (6-mm Gaussian kernel) decoding accuracy maps (minus 50% chance level). For the connectivity analysis, we performed a one-sample *t* test on the difference between parameter estimates of the psychophysiological regressors for inferred and conditioned cues. The PPI results were small volume corrected for FWEs at the voxel level using the HPC ROIs defined above.

All ROI-based decoding results reported here were tested against the empirical chance level estimated for each analysis and subject. To determine empirical chance level, we repeated each analysis 10,000 times with the labels in the test data randomly permuted. Decoding accuracies from these random permutations were averaged and used as empirical chance level per subject. Statistical significance level was *p* < 0.05, one-tailed and two-tailed for directed and undirected hypotheses, respectively. For voxelwise tests, we used small volume correction for FWE at the voxel level using the OFC and HPC ROIs defined above. Effects outside of these ROIs are reported if they survive whole-brain FWE correction.

For repeated measures ANOVA on the behavioral data, Greenhouse–Geisser correction was applied, as appropriate.

## Supporting information

S1 Data Excel spreadsheet containing, in separate sheets, the underlying numerical data for Figure panels 2a, 2b, 2c, 2d, 2e, 2f, 3c, 4b, 4c, 6c, and 6d. (XLSX)

## Abbreviations

d′: discrimination sensitivity
FWE: familywise error
fMRI: functional magnetic resonance imaging
HPC: hippocampus
ISI: interstimulus interval
ITI: intertrial interval
MVPA: multivoxel pattern analysis
OFC: orbitofrontal cortex
PPI: psychophysiological interaction
ROI: region of interest
RT: response time
SVM: support vector machine
vmPFC: ventromedial prefrontal cortex
